# Integrated Transcriptomic and Single-Cell Analyses Identify CCR2-Dependent Monocyte–Smooth Muscle Cell Crosstalk in Aortic Aneurysm

**DOI:** 10.3390/jcdd13100491

**Published:** 2026-09-30

**Authors:** Binbin Zhang, Minnan Gao, Lu Zhang, Yongjia Qiang, Bin Li, Kuan Zeng, Yanqi Yang

**Affiliations:** 1 Biological Laboratory of Hetao Cooperation Zone, the Eighth Affiliated Hospital of Sun Yat-sen University, Shenzhen 518033, China; sci200623@163.com (B.Z.);; 2 Department of Cardiovascular Surgery, The First Affiliated Hospital of Kunming Medical University, Kunming 650032, China

**Keywords:** aortic aneurysm, CCR2, monocyte, vascular smooth muscle cell, single-cell RNA sequencing, transcriptomics, paracrine crosstalk

## Abstract

Background/Objectives: Aortic aneurysm (AA) involves inflammatory recruitment and vascular smooth muscle cell (VSMC) remodeling, but the cellular contribution of candidate chemokine receptors remains unclear. We prioritized reproducible AA-associated genes, localized their cellular sources, and tested whether monocyte CCR2 influences human aortic smooth muscle cells (HASMCs). Methods: Differentially expressed genes shared by GSE57691 and GSE7084 underwent enrichment, protein–protein interaction, regulatory-network, and drug–gene analyses. Hub genes were assessed in GSE9106 and localized in GSE166676 single-cell RNA-sequencing data. CCR2-silenced THP-1 monocytic cells were indirectly co-cultured with HASMCs. CCR2 knockdown was verified by quantitative PCR; HASMC remodeling-related proteins and 24-h scratch-wound closure were evaluated. Results: We identified 107 common upregulated genes and ten immune-related hubs. CCR2 was monocyte-enriched and higher in AA cells overall (*p* = 0.00799) and AA monocytes (*p* = 0.00440). THP-1 co-culture increased COL3A1 and SPP1, decreased α-SMA and TPM, and increased HASMC wound closure. CCR2 silencing partially reversed these changes and decreased wound closure versus siNC-THP-1 cells (*p* < 0.01). Conclusions: Multi-omic evidence and non-contact co-culture support CCR2-dependent monocyte–HASMC paracrine crosstalk in AA-associated remodeling. The responsible mediators and in vivo relevance require further study.

## 1. Introduction

Aortic aneurysm is characterized by progressive aortic dilatation and wall weakening and can culminate in rupture or dissection. Its natural history and global mortality underscore the substantial clinical burden associated with rupture and related complications [1,2]. Imaging surveillance and operative repair remain the principal clinical strategies, but neither directly addresses the molecular processes that initiate and sustain wall degeneration. AA is increasingly understood as a multicellular disorder involving immune-cell infiltration, extracellular-matrix turnover, and plasticity of vascular smooth muscle cells (VSMCs) [3,4]. Oxidative injury, ferroptosis-related responses, and dysregulated TGF-β signaling further contribute to aortic-wall degeneration [5,6].

The biological heterogeneity of aortic aneurysm is reflected in its genetic architecture and non-coding RNA regulation [7,8]. Matricellular proteins and long non-coding RNAs also participate in extracellular-matrix remodeling and vascular-cell state regulation [9,10]. Clinical populations differ by aneurysm location and cardiovascular comorbidity, which complicates the interpretation of mixed aortic cohorts [11,12].

Public transcriptomic datasets can reveal reproducible disease-associated signals across cohorts, and integrative analyses have implicated immune activation and inflammatory signaling in aortic aneurysm [13,14]. Transcriptomic comparisons of inflammatory and noninflammatory aneurysms and bioinformatic studies of inflammaging further support the importance of immune context [15,16]. Lipidomic and dynamic-signaling studies likewise indicate that aneurysmal remodeling is molecularly heterogeneous [17,18]. Yet bulk tissue profiles cannot distinguish changes in cell abundance from cell-state changes. Single-cell RNA sequencing can address this limitation by localizing candidate genes to defined vascular and immune compartments.

CCR2 is a chemokine receptor expressed prominently by circulating and tissue-infiltrating monocytes. Experimental work has linked CCR2-dependent inflammatory-cell recruitment to aneurysm formation, while bone-marrow-derived MCP1/CCL2 can influence VSMC matrix remodeling and phenotypic state [19,20]. However, computational nomination alone does not establish functional communication between CCR2-positive monocytes and aortic smooth muscle cells.

MicroRNA studies have identified regulatory networks and candidate miRNA–mRNA interactions in aortic aneurysm [21,22]. However, computationally predicted regulatory links require experimental validation. Combining bulk, single-cell, and focused functional evidence is therefore especially useful for chemokine receptors, whose tissue-level signal may be driven by infiltrating leukocytes.

**Figure 1:**
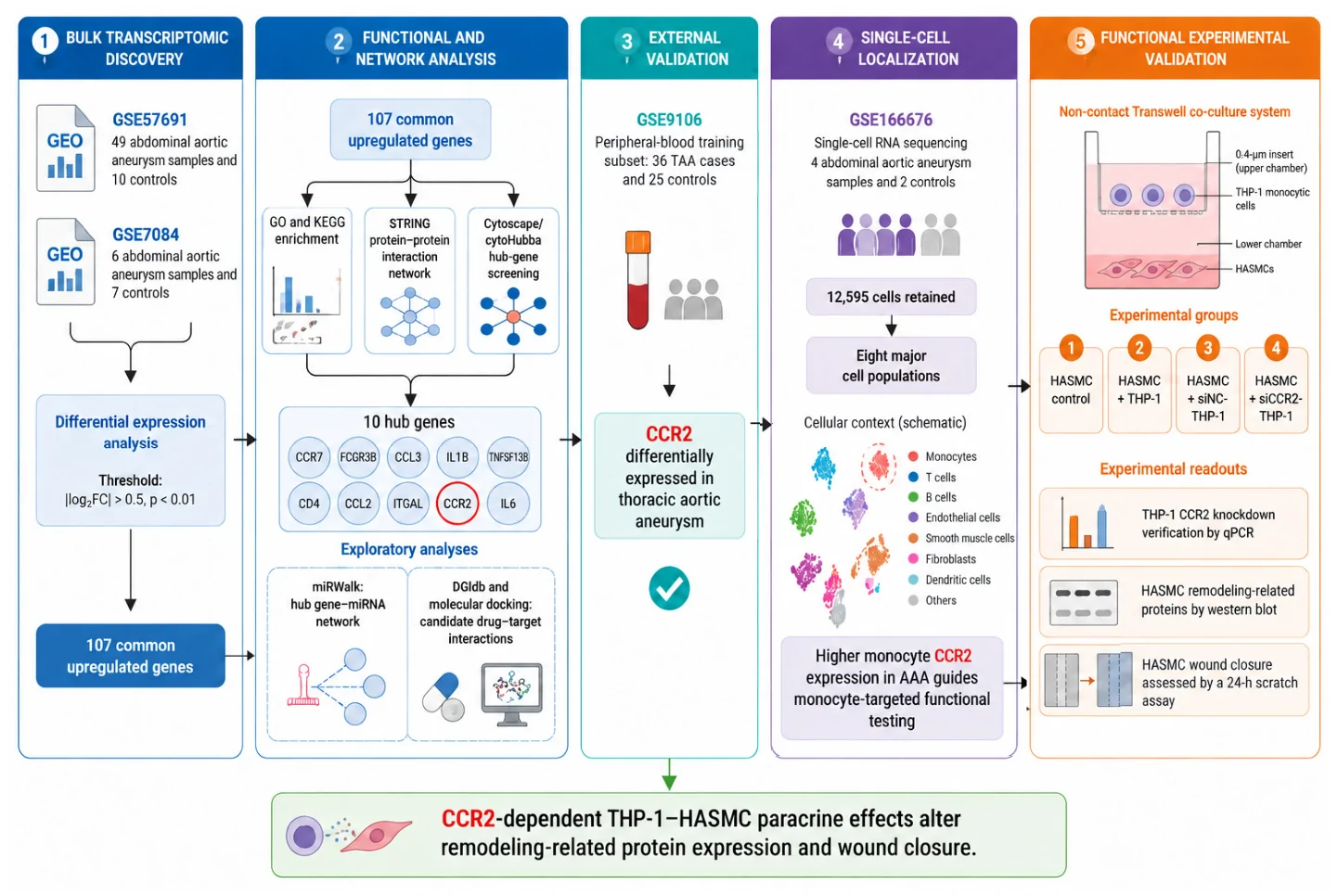
Study design and evidence integration. Abdominal aortic tissue discovery was followed by immune-gene prioritization, external expression assessment in the GSE9106 peripheral-blood TAA training subset (36 cases and 25 controls), and cellular contextualization using GSE166676 (four AAAs and two controls). The single-cell analysis guided monocyte-targeted CCR2 silencing; the functional model assessed HASMC remodeling-related proteins and 24-h wound closure. The figure distinguishes cohort association from functional testing and does not establish a specific ligand–receptor mechanism.

We therefore integrated two discovery transcriptomic cohorts, an external validation dataset, and a single-cell AA dataset to prioritize and localize candidate genes. We then used an indirect THP-1–HASMC co-culture model in which CCR2 was silenced specifically in THP-1 cells. This design asked a focused question: does monocyte CCR2 contribute to paracrine changes in HASMC remodeling-related proteins and 24-h scratch-wound closure? Drug and miRNA analyses were retained as exploratory, hypothesis-generating components. The study workflow is summarized in Figure 1.

## 2. Materials and Methods

### 2.1. Public Datasets and Study Design

This study combined secondary analysis of de-identified public datasets with in vitro cell-culture experiments. The discovery datasets comprised abdominal aortic tissue: GSE57691 included 49 abdominal aortic aneurysm (AAA) samples and 10 organ-donor controls, and GSE7084 included 6 AAA samples and 7 non-aneurysmal abdominal aortic controls [23,24]. The nine aortic occlusive disease samples in the full GSE57691 series were not part of the AAA–control comparison. External expression assessment used the GSE9106 training subset of peripheral blood from 36 thoracic aortic aneurysm (TAA) cases and 25 controls [25]. GSE166676 provided single-cell profiles from four AAA samples and two non-aneurysmal aortic controls [26]. These cohorts differ in anatomical disease location and biospecimen; the blood-based TAA analysis was treated as cross-cohort support rather than direct replication in abdominal aortic tissue. No new human tissue or animal experiment was performed. Dataset characteristics and sample numbers are summarized in Table 1.

### 2.2. Differential Expression Analysis

Differential expression in GSE57691 and GSE7084 was analyzed in R 4.2.1 using limma. Genes with |log2 fold change| > 0.5 and nominal *p* < 0.01 were considered differentially expressed. Common upregulated genes were defined by intersection of the two datasets and visualized by volcano plots, heatmaps, and a Venn diagram.

### 2.3. Enrichment, Protein Interaction, and Hub-Gene Analyses

Gene Ontology and Kyoto Encyclopedia of Genes and Genomes enrichment analyses were performed using clusterProfiler [27]; adjusted *p* < 0.05 defined significant terms. STRING interactions were imported into Cytoscape version 3.10.4 [28] with a confidence threshold of 0.4. Hub genes were ranked using the cytoHubba plug-in.

### 2.4. miRNA and Drug–Gene Analyses

Exploratory hub-gene–miRNA links were queried in miRWalk (prediction score > 0.95; connections to at least four hub genes), and drug–gene links were retrieved from DGIdb [29]. These analyses generated hypotheses and were not used to support the co-culture mechanism or therapeutic efficacy.

### 2.5. External Transcriptomic Validation

Hub-gene expression was assessed in the GSE9106 training subset comprising 36 thoracic aortic aneurysm cases and 25 controls. Between-group tests were performed in R after inspection of distributional assumptions; two-sided *p* < 0.05 was considered significant.

### 2.6. Single-Cell RNA-Sequencing Analysis

Raw 10× Genomics data from GSE166676 were processed using Seurat. Cells passing quality-control filters were log-normalized, highly variable features were selected, and principal-component analysis was performed. Harmony was used to reduce sample-level batch effects. Clustering used the first 15 Harmony dimensions at resolution 0.5. Major populations were annotated using canonical markers, including *PTPRC*, *CD3D*/*CD3E*/*CD4*, *MS4A1*/*CD79A*/*CD19*, *NKG7*/*KLRB1*, *CD14*/*ITGAM*, *CD68*/*CD163*, *VWF*, *ACTA2*/*MYH11*, and *EPCAM*/*KRT19*. Between-condition comparisons used Wilcoxon rank-sum tests.

### 2.7. Molecular Docking

Exploratory docking used Protein Data Bank target structures, PubChem ligands, and AutoDock Vina version 1.1.2; the lowest predicted binding energy was recorded for computational prioritization only.

### 2.8. Cell Culture and Indirect Co-Culture

Human THP-1 monocytic cells were obtained from Beyotime Biotechnology (Shanghai, China; Cat. No. C6960) and maintained in RPMI 1640 medium supplemented with 10% fetal bovine serum (FBS). The immortalized human aortic smooth muscle cell line (HASMC) was obtained from Zhejiang Baidi Biotechnology Co., Ltd. (BDBIO, Hangzhou, China; Cat. No. C6555) and cultured as adherent cells in DMEM supplemented with 10% FBS. According to the supplier documentation, the HASMC line was established from primary human aortic smooth muscle cells by SV40T-mediated immortalization and characterized by the supplier using α-SMA immunofluorescence. The documentation identifies the tissue source as human aorta but does not specify the anatomical segment. For indirect co-culture in six-well plates, HASMCs were seeded in the lower chamber at 3 × 10^5^ cells/well and THP-1 cells in 0.4-μm-pore upper inserts at 1 × 10^6^ cells/insert, allowing exchange of soluble factors without direct cell contact. DMEM supplemented with 0.5% FBS was used during co-culture. Four conditions were examined: HASMC monoculture control, HASMCs co-cultured with untreated THP-1 cells, HASMCs co-cultured with siNC-transfected THP-1 cells, and HASMCs co-cultured with *siCCR2*-transfected THP-1 cells. Co-culture was continued for 24 h before HASMC collection and functional analysis.

### 2.9. CCR2 Silencing in THP-1 Cells

THP-1 cells were transfected with a CCR2-targeting small interfering RNA (*siCCR2*; Tsingke Biotechnology, Beijing, China) or a non-targeting control (siNC) using NanoTrans™ Transfection Reagent 3000 (CYTOCH, Shanghai, China; Cat. No. CT0006-1.5ML). The *siCCR2-2* duplex (Tsingke order GZP26041600007; product H729230-SICCR2-2) comprised the sense strand 5′-CAACGAGAGCGGUGAAGAA-dTdT-3′ and antisense strand 5′-UUCUUCACCGCUCUCGUUG-dTdT-3′. The final siRNA concentration was 50 nM. Co-culture was initiated 24 h after transfection. CCR2 knockdown was assessed in THP-1 cells by quantitative reverse-transcription PCR.

### 2.10. RNA Isolation and Quantitative PCR

Total RNA was isolated from THP-1 cells using the Eastep Super Total RNA Extraction Kit (Promega, Shanghai, China; Cat. No. LS1040) and reverse-transcribed using the Reverse Transcription Kit (with dsDNase; Huayunbio, Guangzhou, China; Cat. No. HYR323). Quantitative PCR was performed using Universal SYBR qPCR Master Mix (Huayunbio, Guangzhou, China; Cat. No. HBQ711) on a CFX96 Touch Real-Time PCR Detection System (Bio-Rad, Hercules, CA, USA). β-actin (*ACTB*) was used as the reference gene. The *CCR2* primers were 5′-CAGGTGACAGAGACTCTTGGGA-3′ (forward) and 5′-GGCAATCCTACAGCCAAGAGCT-3′ (reverse). The β-actin primers were 5′-CACCATTGGCAATGAGCGGTTC-3′ (forward) and 5′-AGGTCTTTGCGGATGTCCACGT-3′ (reverse). Reactions were run in a 20-μL volume with an initial 95 °C step for 5 min, followed by 40 cycles of 95 °C for 10 s and 60 °C for 30 s with fluorescence acquisition. This was followed by 95 °C for 30 s and 60 °C for 30 s, then a melt curve from 70 °C to 95 °C in 0.5 °C increments with a 10-s hold per step. Oligonucleotide sequences are summarized in Table S4. Relative *CCR2* expression was calculated using the comparative-cycle method and normalized to control. Three independent experiments were performed, with three technical PCR replicates per sample. Technical replicates were not treated as independent biological observations.

### 2.11. Western Blotting

After 24 h of co-culture, lower-chamber HASMCs were harvested for immunoblotting of COL3A1, SPP1, α-SMA, and TPM, with β-actin as the loading control. Band intensities were quantified by densitometry, normalized to β-actin, and summarized from three independent experiments.

### 2.12. Scratch-Wound Closure Assay

Confluent HASMC monolayers were scratched uniformly, washed, and imaged at baseline. Co-culture was initiated immediately after scratching, and the wounds were imaged again 24 h later. The assay was performed in medium containing 0.5% FBS without an antiproliferative treatment. Wound area was quantified using identical settings across groups. Wound closure at 24 h was calculated as (wound area at 0 h − wound area at 24 h)/wound area at 0 h × 100%. Three independent experiments were performed, with three replicate wells per condition in each experiment. Three fields per well were analyzed, and the well/field measurements were aggregated into one value per condition for each independent experiment. Replicate wells and imaging fields were subsamples rather than additional independent experiments. This endpoint does not isolate migration from proliferation or cell survival.

### 2.13. Statistical Analysis

Data are presented as mean ± standard deviation from three independent experiments. CCR2 qPCR and the 24-h scratch-wound closure were compared by one-way analysis of variance followed by Tukey multiple-comparison tests. Band intensities from Western blots were quantified by densitometry from three independent experiments and normalized to β-actin. A two-sided *p* < 0.05 was considered statistically significant.

## 3. Results

### 3.1. Common Differentially Expressed Genes

**Figure 2:**
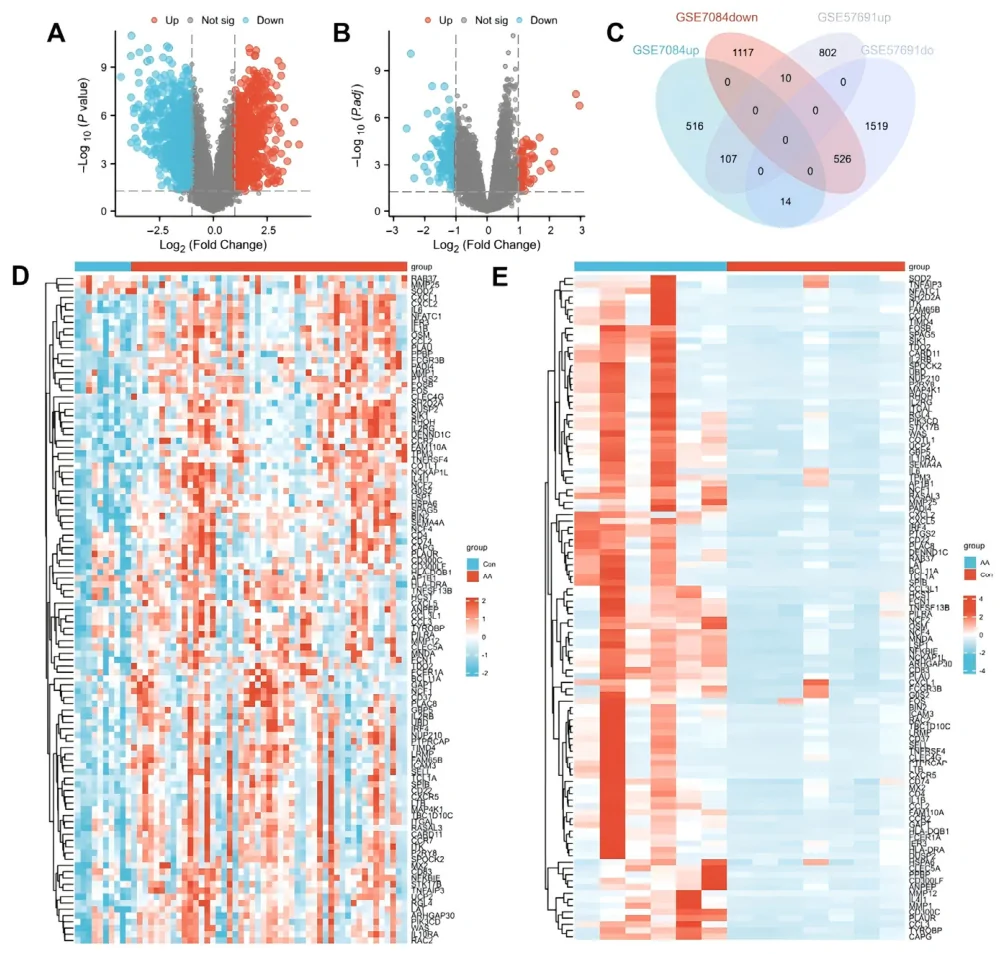
Differentially expressed genes in GSE7084 and GSE57691. (**A**,**B**) Volcano plots. (**C**) Intersection of differentially expressed genes. (**D**,**E**) Heatmaps of common genes. Thresholds were |log2FC| > 0.5 and *p* < 0.01.

GSE7084 yielded 516 upregulated and 1117 downregulated genes, whereas GSE57691 yielded 802 upregulated and 1519 downregulated genes. Intersection identified 107 genes upregulated in both datasets (Figure 2).

### 3.2. Immune and Chemokine Pathways Predominate

**Figure 3:**
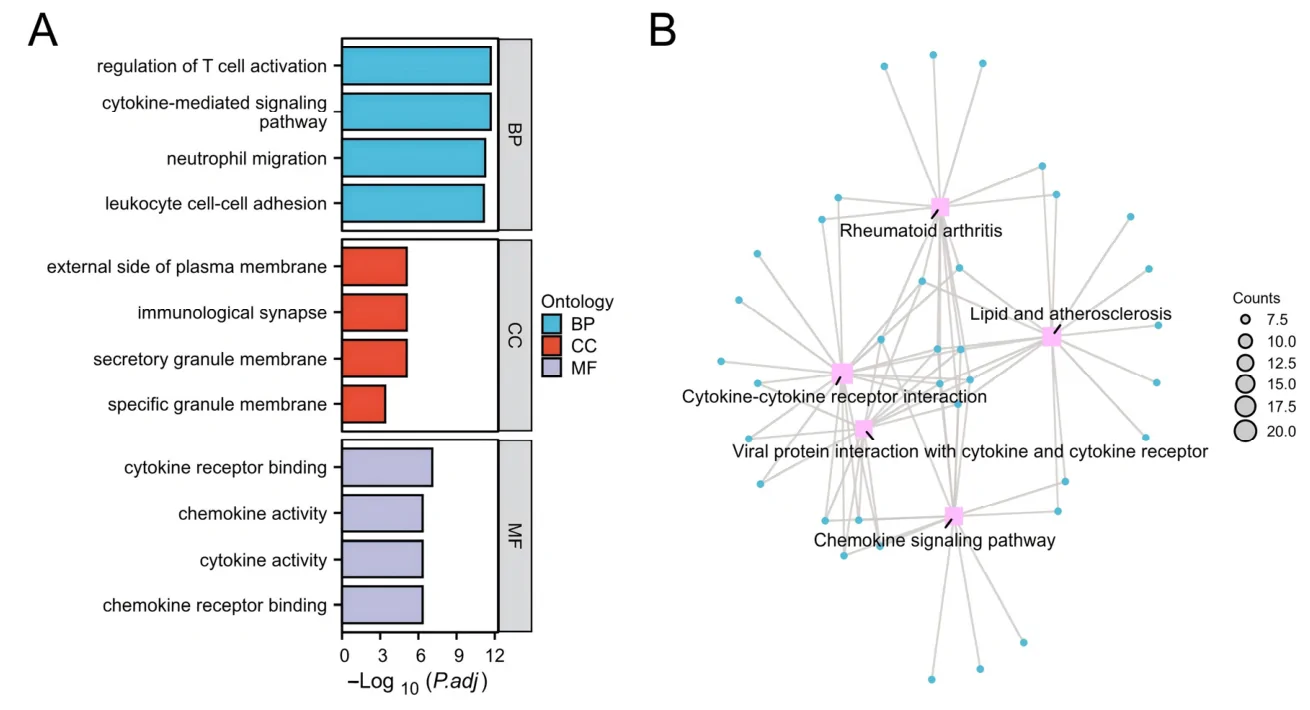
Gene Ontology and KEGG enrichment of the 107 common upregulated genes. (**A**) Gene Ontology categories. (**B**) Gene–pathway network for selected KEGG terms.

The 107 common upregulated genes were enriched for T-cell activation, cytokine-mediated signaling, neutrophil migration, leukocyte adhesion, cytokine/chemokine activity, cytokine–cytokine receptor interaction, and chemokine signaling (Figure 3).

### 3.3. Network Analysis Prioritizes Ten Hub Genes

**Figure 4:**
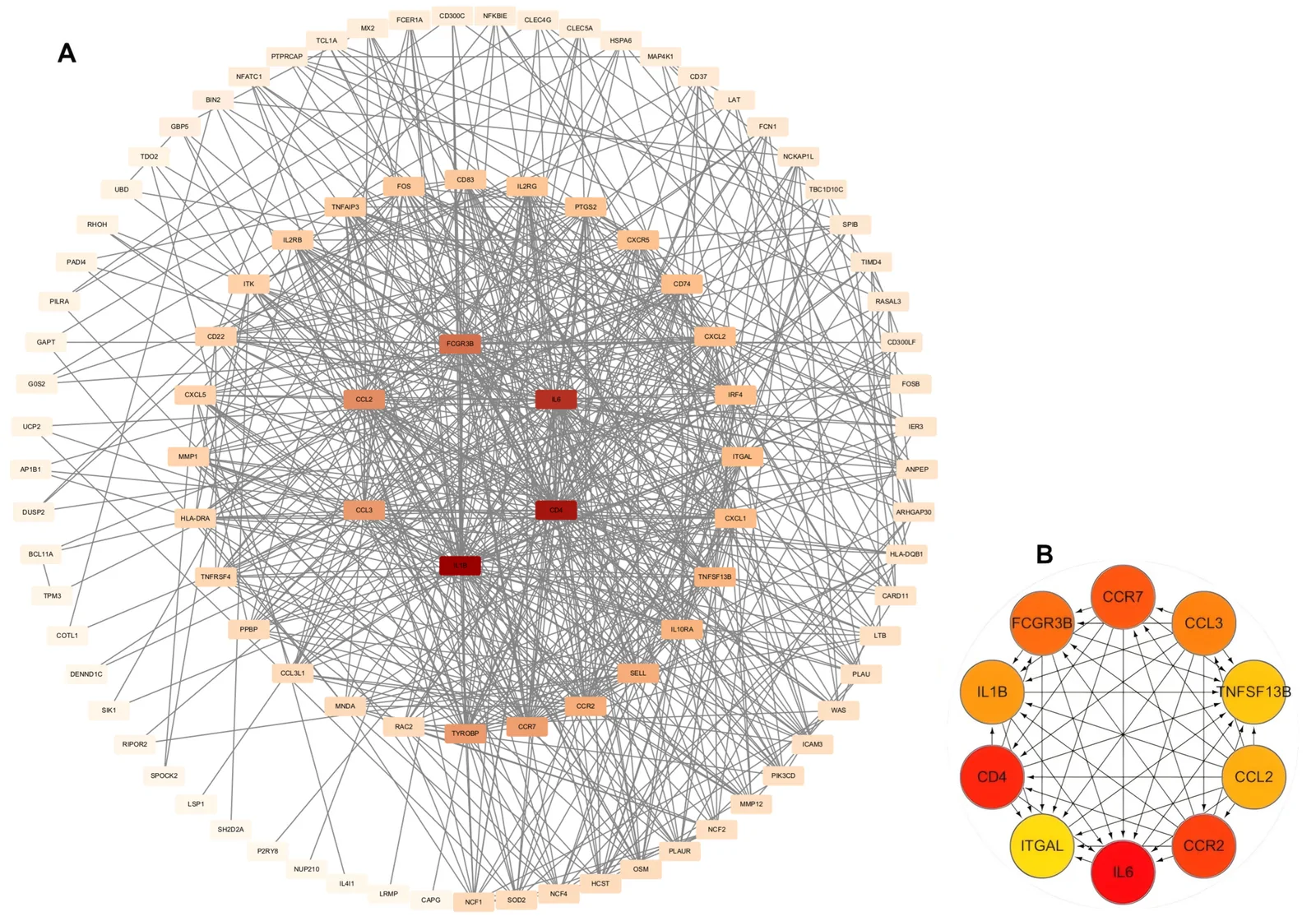
Protein–protein interaction and hub-gene analyses. (**A**) STRING-derived interaction network. (**B**) Ten hub genes ranked in Cytoscape.

The STRING network contained 107 nodes and 653 edges. CytoHubba prioritized *CCR7*, *FCGR3B*, *CCL3*, *IL1B*, *TNFSF13B*, *CD4*, *CCL2*, *ITGAL*, *CCR2*, and *IL6* (Figure 4).

### 3.4. Exploratory Drug and miRNA Networks

**Figure 5:**
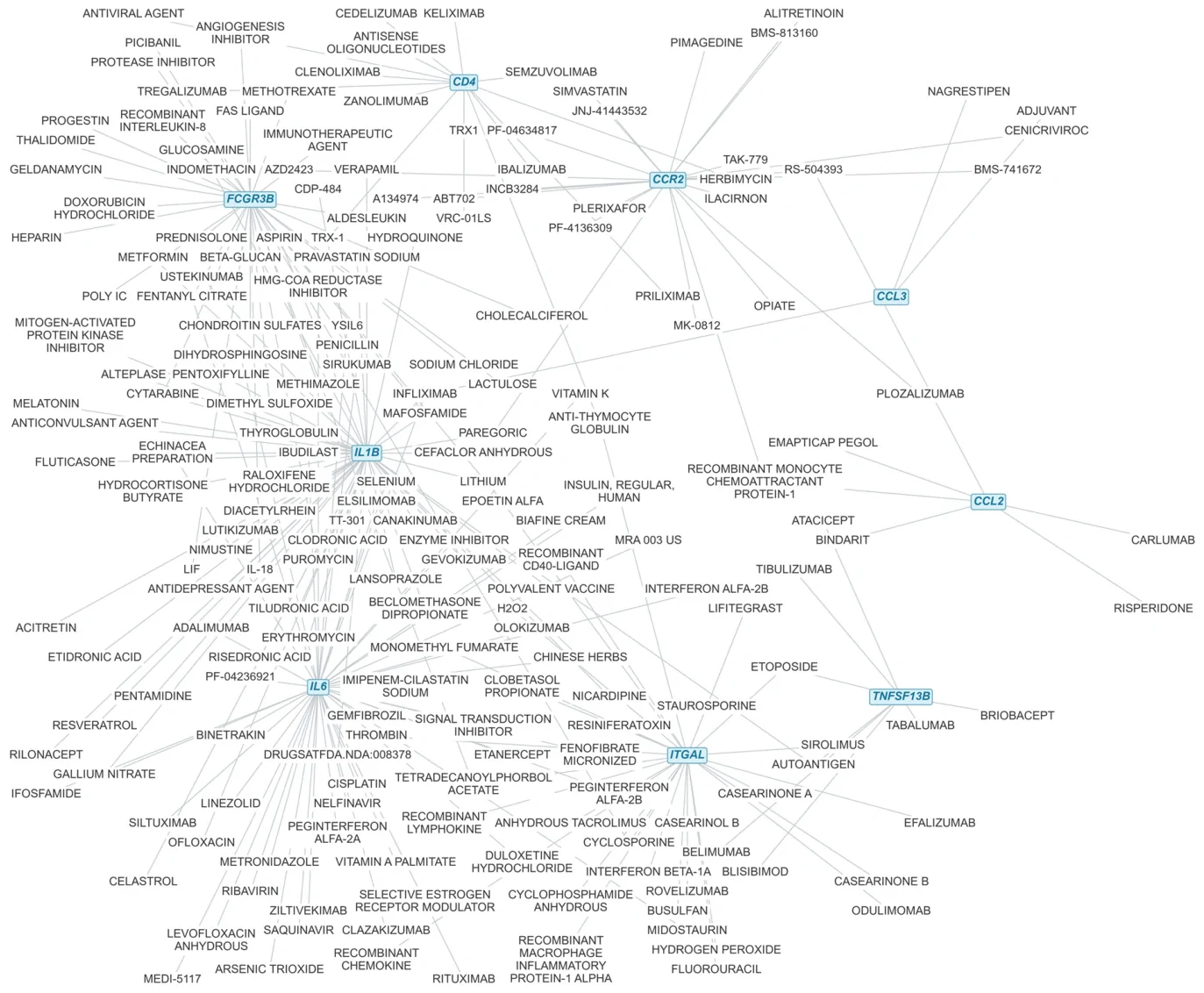
Exploratory drug–gene interaction network. Blue labels indicate hub genes and black labels indicate drugs or compounds.

**Figure 6:**
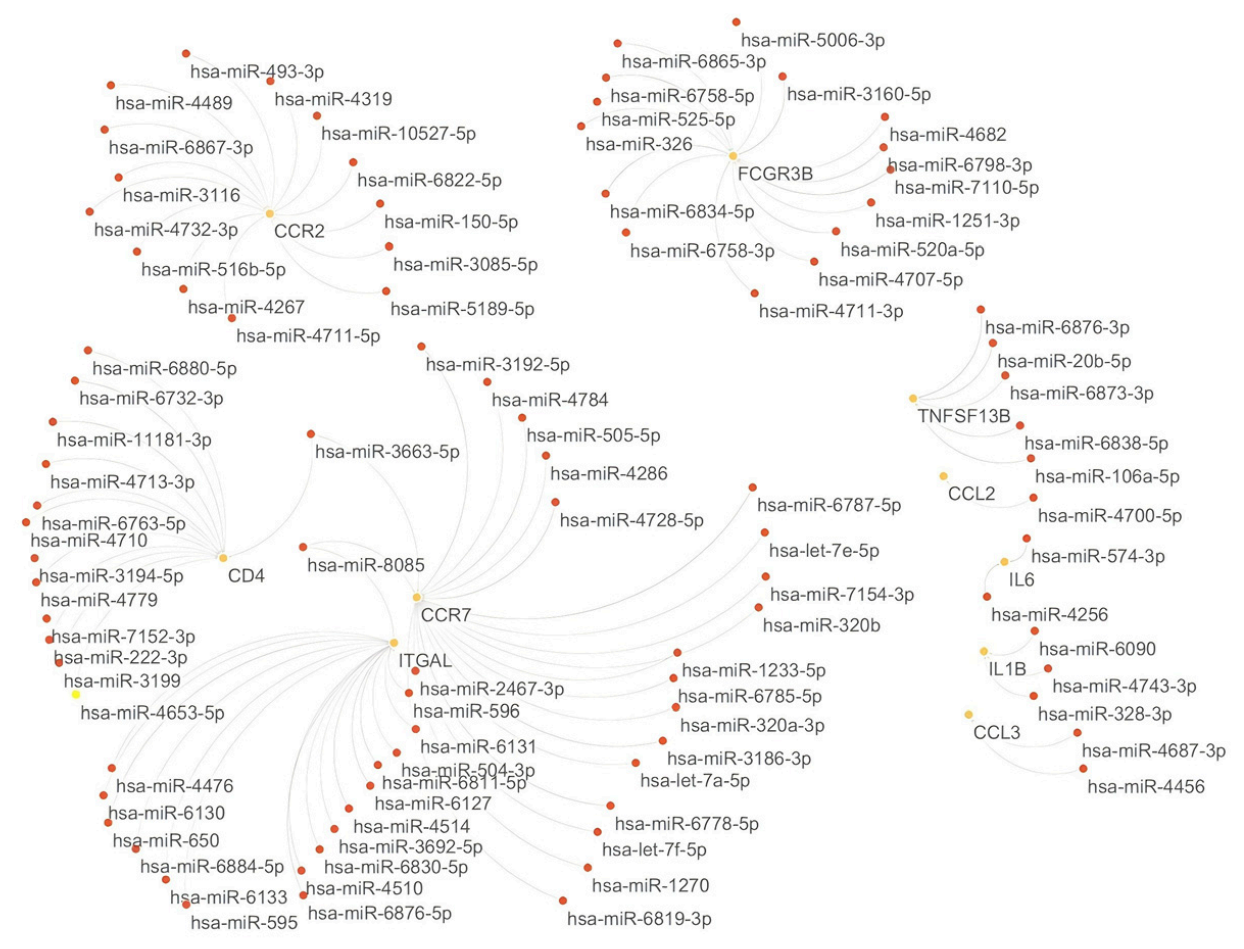
Predicted hub-gene–miRNA interaction network. Yellow nodes indicate hub genes and red nodes indicate predicted miRNAs.

DGIdb and miRWalk generated exploratory drug and miRNA networks, respectively (Figure 5 and Figure 6); the latter contained 91 predicted miRNAs. These predictions were not functionally validated.

### 3.5. External Dataset Supports CCR2

**Figure 7:**
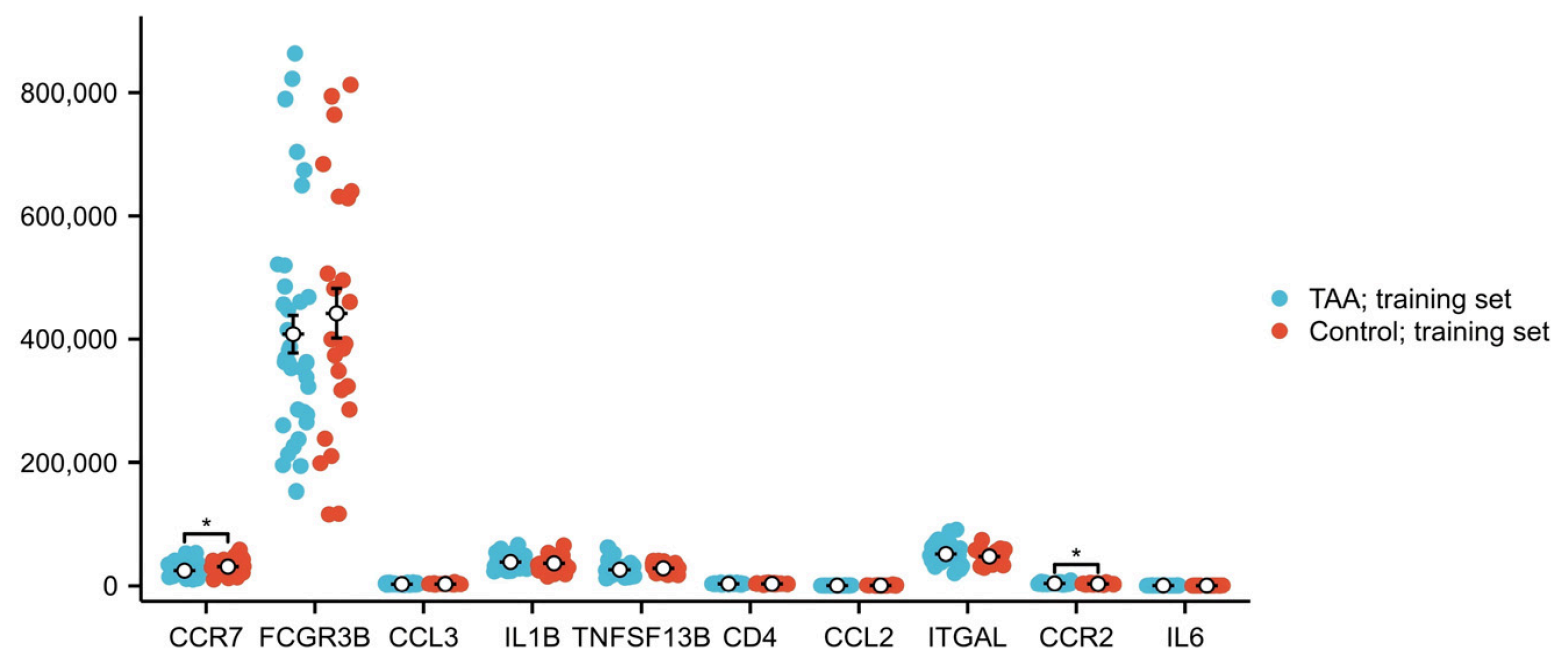
Hub-gene expression in the GSE9106 peripheral-blood training subset (36 TAA cases and 25 controls). *CCR2* and *CCR7* differed between groups (* *p* < 0.05). The analysis evaluates a distinct anatomical disease category and biospecimen from the abdominal aortic discovery datasets.

In the analyzed GSE9106 subset, *CCR2* and *CCR7* differed between TAA and control samples (both *p* = 0.0373); the other eight hub genes were not significant (Figure 7). *CCR2 was higher in the TAA group*, whereas *CCR7* was lower.

### 3.6. Single-Cell Analysis Localizes CCR2 to Monocytes

**Figure 8:**
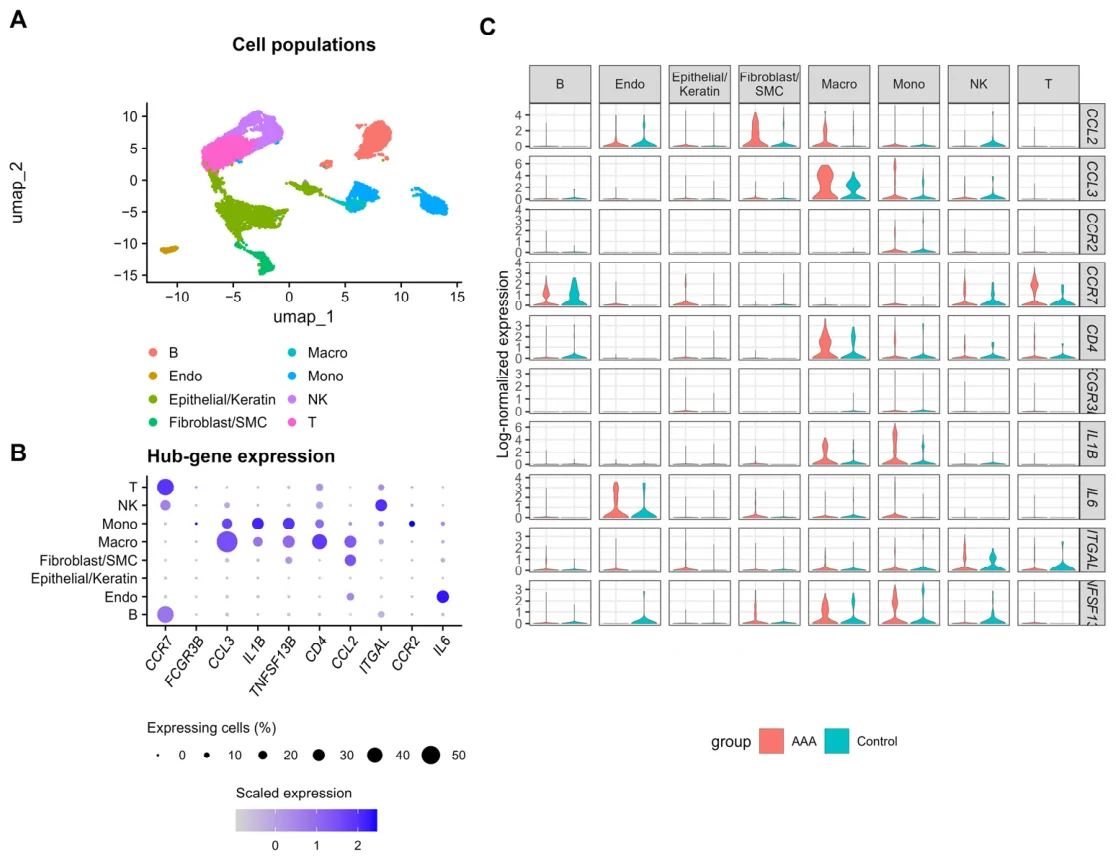
Single-cell analysis of GSE166676: four AAA samples and two non-aneurysmal aortic controls, comprising 12,595 retained cells. (**A**) UMAP annotation of major populations. (**B**) Hub-gene expression across populations. (**C**) Between-condition expression comparisons within cell types. Wilcoxon comparisons were performed at the cell level and are exploratory; cells do not represent additional independent donors.

After quality control and integration, 12,595 cells from four AAA samples and two controls formed 18 clusters and eight major annotated populations. *CCR2* expression was higher in AAA cells overall (*p* = 0.00799; log2FC = 0.337) and localized mainly to monocytes, where it was also higher in AAA than control cells (*p* = 0.00440; log2FC = 0.424). These exploratory cell-level comparisons provide disease-associated cellular context for the bulk signal and motivated *CCR2* perturbation in THP-1 cells. *CCL2* and *TNFSF13B* signals were observed in the fibroblast/VSMC compartment, but expression patterns alone do not establish communication between these populations (Figure 8).

### 3.7. Docking Prioritizes MK-0812 as a CCR2-Binding Candidate

**Figure 9:**
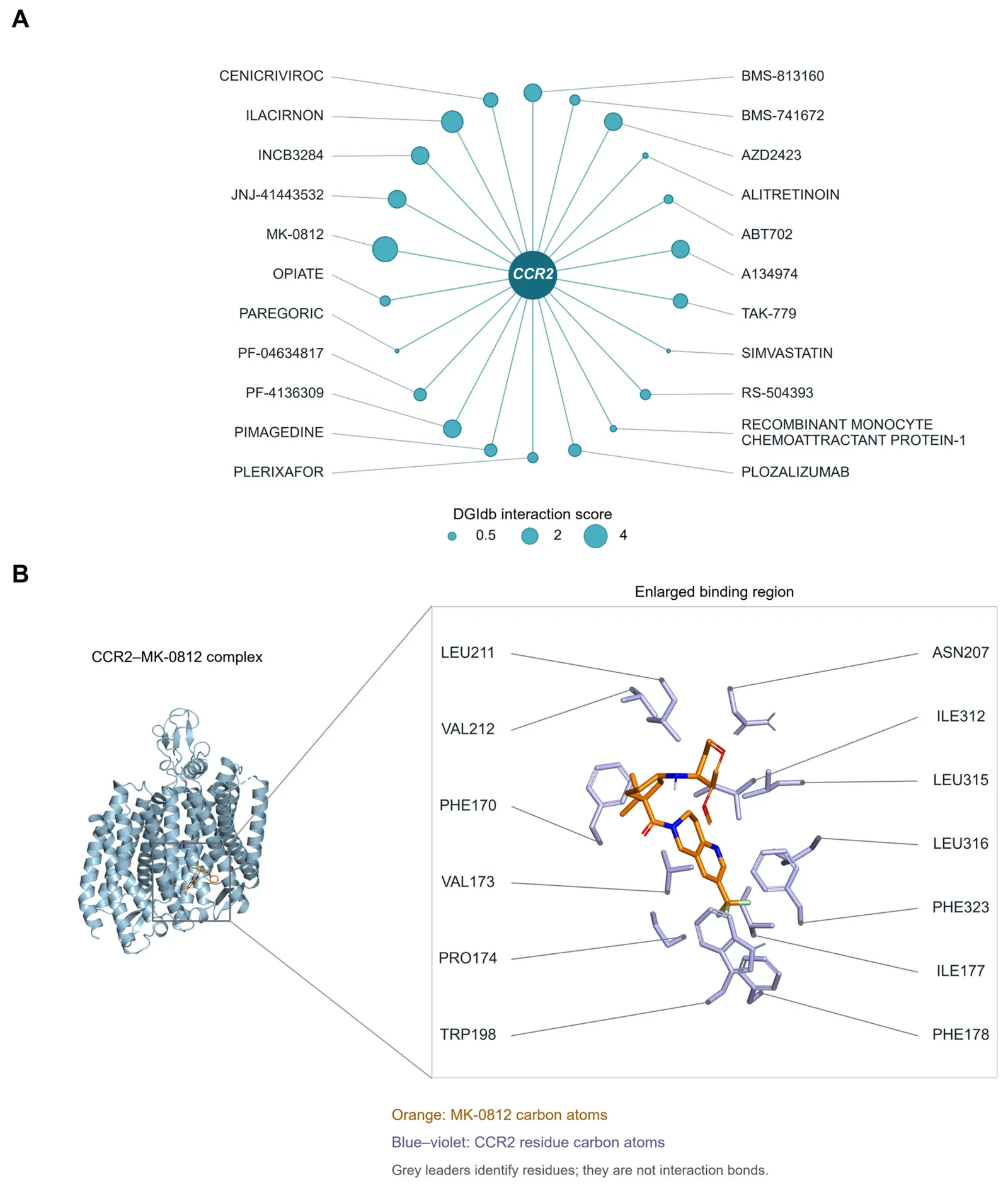
Exploratory drug screening and molecular docking. (**A**) Candidate drug–hub-gene network. (**B**) Predicted CCR2–MK-0812 binding pose. An overview and a close-up of nearby residues are shown in panel B.

MK-0812 had the most favorable predicted CCR2 binding energy among screened pairs (−8.1 kcal/mol; Figure 9), without experimental evidence of target engagement or efficacy.

### 3.8. THP-1 CCR2 Silencing Attenuates Co-Culture-Induced HASMC Changes

**Figure 10:**
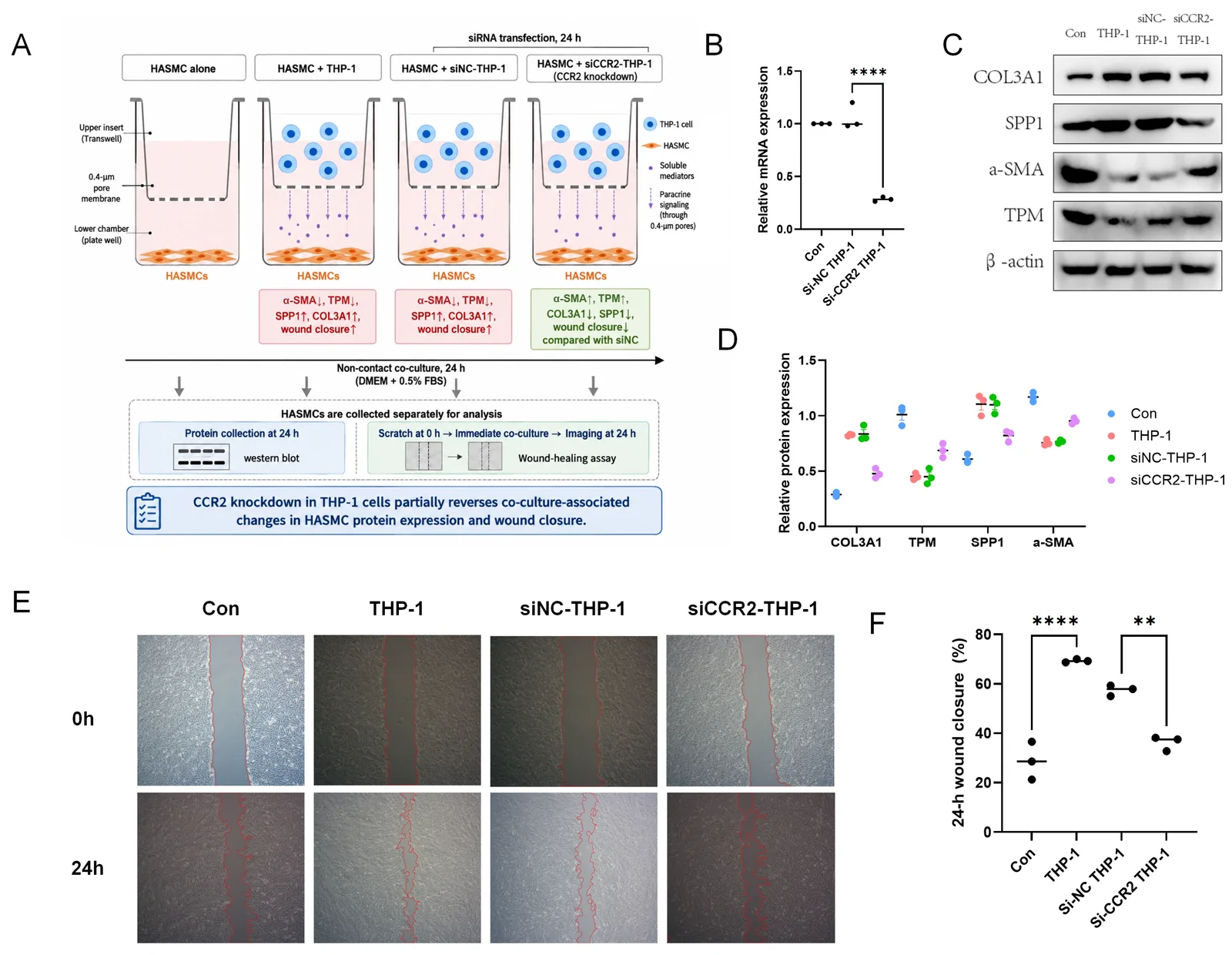
Experimental evaluation of CCR2-dependent THP-1–HASMC paracrine effects. (**A**) Experimental design. THP-1 cells were transfected 24 h before co-culture. For the scratch assay, HASMCs were scratched immediately before co-culture, maintained in DMEM with 0.5% FBS without antiproliferative treatment, and imaged at 0 and 24 h. (**B**) CCR2 mRNA knockdown in THP-1 cells, measured by qPCR. (**C**) Representative HASMC Western blots of COL3A1, SPP1, α-SMA, and TPM, with β-actin as loading control. (**D**) Densitometric quantification. (**E**) Representative scratch-wound images at 0 and 24 h. (**F**) Wound closure at 24 h. Each point in (**B**,**D**,**F**) represents one of three independent experiments per group; horizontal markers indicate group means, and error bars in (**D**) indicate SD. qPCR used three technical replicates per sample. Scratch experiments used three wells per condition and three fields per well; these measurements were aggregated into one value per independent experiment. Group colors are identified in the legend of (**D**); (**B**,**F**) use black symbols. Brackets indicate the comparisons shown: ** *p* < 0.01; **** *p* < 0.0001, from one-way ANOVA followed by Tukey tests in (**B**,**F**). Technical replicates and fields were not counted as independent experiments.

*CCR2* mRNA expression was markedly reduced in *siCCR2*-transfected THP-1 cells compared with siNC-transfected THP-1 cells (0.285 ± 0.021 versus 1.060 ± 0.123; *n* = 3 independent experiments; *p* < 0.0001; Figure 10B), confirming efficient transcript-level knockdown.

Densitometric analysis of three independent Western blot experiments showed that THP-1 and siNC-THP-1 co-culture increased COL3A1 and SPP1 abundance and decreased α-SMA and TPM abundance in HASMCs compared with monoculture. CCR2 knockdown in THP-1 cells partially reversed these changes: COL3A1 and SPP1 decreased, whereas α-SMA and TPM increased relative to the siNC-THP-1 group (Figure 10C,D). Individual replicate values are provided in the accompanying source-data file.

At 24 h, THP-1 co-culture increased HASMC wound closure relative to monoculture (*p* < 0.0001). CCR2 silencing in THP-1 cells decreased HASMC wound closure relative to the siNC-THP-1 group (*p* < 0.01; Figure 10E,F). These results support a CCR2-dependent effect of THP-1 cells on HASMC scratch-wound closure. Individual replicate values are provided in the accompanying source-data file.

## 4. Discussion

The present study combines cross-cohort transcriptomic discovery, external validation, single-cell localization, and targeted cell experiments to identify CCR2-associated monocyte–HASMC paracrine effects in aortic aneurysm. The common upregulated genes were dominated by immune- and chemokine-related functions, consistent with prior studies showing inflammatory pathway activation and immune-cell infiltration in aortic aneurysm [14,15]. CCR2 was supported in an independent bulk dataset and localized predominantly to monocytes in the single-cell dataset. The co-culture experiments then provided functional evidence that CCR2 expression in THP-1 monocytic cells contributes to paracrine changes in HASMC protein expression and scratch-wound closure. Thus, the main advance is not simply the identification of another differentially expressed gene, but the connection of a tissue-level CCR2 signal to a plausible monocyte–smooth muscle cell interaction.

The monocyte localization of CCR2 is biologically consistent with the established role of this receptor in inflammatory-cell recruitment. In an experimental aneurysm model, genetic deletion of CCR2 reduced inflammatory-cell accumulation, preserved aortic wall architecture, decreased MMP2 and MMP9 activity, and attenuated aneurysm formation [19]. Macrophage-centered inflammatory programs have likewise been shown to influence experimental aortic aneurysm progression [30]. Our single-cell results extend this evidence by indicating that the increased CCR2 signal in human aneurysm tissue is concentrated mainly in monocytes. Nevertheless, increased expression may reflect both greater monocyte abundance and altered activation within the monocyte compartment. The present data cannot fully separate these two possibilities.

The final Western blot results support a CCR2-dependent remodeling response in HASMCs. THP-1 and siNC-THP-1 co-culture reduced α-SMA and TPM while increasing SPP1 and COL3A1. Loss of contractile markers together with increased SPP1 is compatible with a shift away from a differentiated contractile state, whereas increased COL3A1 suggests altered extracellular-matrix production. VSMC plasticity and loss of contractile-marker expression are recognized features of aneurysmal aortic remodeling [3,4]. Experimental and human aneurysm tissues have also shown early reductions in α-SMA and SM22α before or during structural deterioration [31]. RelB/miR-193a-5p signaling has independently been linked to VSMC phenotypic transformation and migration in aortic aneurysm models [32]. Importantly, CCR2 knockdown in THP-1 cells shifted all four proteins toward the monoculture pattern, linking the HASMC response to CCR2 activity in the monocytic compartment.

These protein changes should still be interpreted as evidence of remodeling rather than proof of a single, fully defined VSMC phenotype. VSMC state transitions are multidimensional and cannot be classified reliably by one or two markers alone [3]. SPP1 elevation supports an osteopontin-associated modulated state, but COL3A1 upregulation may represent matrix synthesis, compensatory repair, or maladaptive fibrosis depending on context. Matricellular and extracellular-matrix proteins can exert context-dependent effects during aneurysmal remodeling [9]. Similarly, the TPM signal provides cytoskeletal information but does not by itself define a contractile lineage state. Additional markers such as MYH11, TAGLN/SM22α, CNN1, MMP2, and proliferation-related proteins, together with transcriptomic or immunofluorescence analysis, would allow more precise phenotypic assignment.

The scratch-wound experiment adds a functional dimension to the protein findings. THP-1 co-culture increased 24-h HASMC wound closure, whereas CCR2 silencing in THP-1 cells attenuated this increase. The assay was performed with 0.5% FBS and without an antiproliferative treatment. Wound closure can reflect migration, proliferation, cell survival, and initial wound geometry; these contributions cannot be separated by the present experiment. The finding therefore supports an effect on collective wound closure rather than a specific effect on migration or chemotaxis. Direct migration assays, proliferation measurements, and live-cell tracking would be needed to distinguish the underlying cellular processes.

The present results are compatible with, but do not directly establish, a CCL2–CCR2 mechanism. Bone-marrow-derived MCP1/CCL2 is required for aneurysm formation in an elastase model and can directly reduce smooth muscle contractile proteins while increasing matrix metalloproteinases [20]. CCL2 was also identified among the hub genes in our bulk analysis, making it a plausible mediator of monocyte–HASMC communication. Nevertheless, the current co-culture experiment manipulated CCR2 only in THP-1 cells. It did not measure secreted CCL2, neutralize CCL2, add recombinant ligand, or perform a ligand-rescue experiment. Other CCR2 ligands or secondary cytokines released after CCR2 activation could therefore account for the observed HASMC response. The mechanistic conclusion should remain limited to CCR2-dependent paracrine crosstalk.

The exploratory miRNA predictions should be interpreted in the context of reported variation in aneurysm miRNA signatures [33,34]. Previous expression-based predictions likewise require functional validation [21,35]. These analyses remain secondary to the monocyte–HASMC experiments.

Drug-network and docking results are hypothesis-generating, consistent with earlier computational compound prioritization [36]. Proteomic studies similarly illustrate the promise and uncertainty of omics-derived candidates [37,38]. No compound was tested here, and the results do not establish pharmacological or clinical efficacy.

Several limitations define the boundaries of the study. First, the abdominal aortic discovery and single-cell cohorts differ from the blood-based TAA validation cohort in anatomical disease category and biospecimen. Shared signals may therefore reflect systemic immune changes rather than a common segment-specific aortic mechanism. The single-cell cohort contains only four AAA samples and two controls, and cell-level comparisons do not constitute donor-level replication. Second, THP-1 cells and the SV40T-immortalized HASMC line may not fully reproduce primary-cell behavior. The HASMC supplier does not specify the aortic segment of origin; consequently, the co-culture findings cannot be interpreted as segment-specific validation of abdominal aortic aneurysm. Third, CCR2 knockdown was verified at the mRNA level, and protein-level confirmation would strengthen target-engagement evidence. Fourth, the non-contact co-culture system demonstrates soluble-factor communication but does not identify the responsible mediator. Fifth, Western blot and scratch-wound analyses were based on three independent experiments, and the latter cannot isolate migration from proliferation. Finally, the study lacks spatial validation in human aneurysm tissue and in vivo intervention. These limitations preclude claims of clinical biomarker performance or therapeutic efficacy.

In summary, the convergence of bulk transcriptomics, single-cell localization, and targeted co-culture experiments supports a model in which CCR2-positive monocytic cells influence aortic smooth muscle cell remodeling through soluble signals. The partial reversal of α-SMA, TPM, SPP1, COL3A1, and wound-closure changes after CCR2 knockdown strengthens receptor dependence, while the unresolved ligand and downstream pathway remain important open questions. Future studies combining primary human monocytes, secreted-factor profiling, ligand neutralization or rescue, spatial tissue validation, and pharmacological intervention will be required to determine whether this axis is mechanistically and therapeutically relevant in aortic aneurysm.

## 5. Conclusions

Integrated bulk and single-cell transcriptomics identify monocyte-enriched CCR2 as a reproducible AA-associated candidate. In the non-contact co-culture model, THP-1 cells decreased α-SMA and TPM, increased SPP1 and COL3A1, and increased HASMC wound closure; CCR2 knockdown in THP-1 cells partially reversed these changes. The results support CCR2-dependent monocyte–smooth muscle cell paracrine communication while leaving the responsible ligand, downstream mediators, and in vivo relevance unresolved.

## Figures and Tables

**Table 1:** Public datasets, anatomical disease categories, biospecimens, and sample numbers included in the analyses. AAA, abdominal aortic aneurysm; TAA, thoracic aortic aneurysm; AOD, aortic occlusive disease. The GSE9106 training subset was the only GSE9106 subset used here.

Dataset	Cohort	Role
GSE57691	Abdominal aortic tissue: 49 AAA and 10 organ-donor controls; excludes 9 AOD samples	Discovery
GSE7084	Abdominal aortic tissue: 6 AAA and 7 non-aneurysmal controls (GPL2507 individual-sample subset)	Discovery
GSE9106	Peripheral blood: TAA training subset, 36 cases and 25 controls	External validation
GSE166676	Aortic scRNA-seq: 4 AAA and 2 non-aneurysmal controls; 12,595 retained cells	Cell-type localization

## Data Availability

The public datasets analyzed in this study are available in the NCBI Gene Expression Omnibus under accession numbers GSE57691, GSE7084, GSE9106, and GSE166676. Numerical source data for the cell experiments and the uncropped Western blot image are provided in the Supplementary Materials. Additional analysis code is available from the corresponding author upon reasonable request.

## Supplementary Materials

The following supporting information can be downloaded at: https://www.mdpi.com/article/10.3390/jcdd13100491/s1, File S1: uncropped Western blot image; Table S1: source data for CCR2 qPCR; Table S2: source data for Western blot densitometry; Table S3: source data for the 24-h scratch-wound assay; Table S4: qPCR primer and *siCCR2-2* sequences.

## Abbreviations

**Abbreviation**	**Definition**
AA	aortic aneurysm
CCR2	C-C motif chemokine receptor 2
DEG	differentially expressed gene
GEO	Gene Expression Omnibus
HASMC	human aortic smooth muscle cell
scRNA-seq	single-cell RNA sequencing
VSMC	vascular smooth muscle cell
